# Is There Any Role of Inhalational Corticosteroids in the Prophylaxis of Post-Traumatic Fat Embolism Syndrome?

**DOI:** 10.7759/cureus.332

**Published:** 2015-09-25

**Authors:** Amit Kumar Agarwal, Ramesh Sen, Sujit K Tripathy, Sameer Aggarwal, Nirmalraj G., Dheeraj Gupta

**Affiliations:** 1 Orthopaedics, Indraprastha Apollo Hospital; 2 Orthopaedics, PGIMER; 3 Orthopaedics, AIIMS, Bhubaneswar; 4 Pulmonary Medicine, PGIMER

**Keywords:** fat embolism syndrome, inhalational steroid, ciclesonide

## Abstract

Fat embolism syndrome (FES) is primarily a lung parenchymal disorder resulting from interstitial and alveolar inflammation triggered by the lipid metabolites in blood circulation. The 'low-dose' corticosteroid is supposed to have a prophylactic effect on the incidence of the FES and arterial hypoxemia by reducing this inflammatory response. It is expected that inhaled corticosteroids (ciclesonide aerosol) may prevent the development of hypoxemia or fat embolism syndrome in high-risk patients by reducing this inflammatory response. Metered-dose inhaler (MDI) steroid preparations can reach the lung parenchyma with minimal systemic effect. Sixty cases of polytrauma patients presenting within eight hours of injury were randomly allocated into one of the two groups. In Group 1 (n_1_=30) ciclesonide, 640 mcg, was given with a metered dose inhaler and repeated once again after 24 hours, whereas Group 2 (n_2_=30) was taken as control and observed for 72 hours for any episode of hypoxia. The outcome was assessed using Schonfeld’s criteria for the eventual outcome of subclinical or clinical FES. Out of 30 patients in each group, six patients developed subclinical FES, whereas three from ciclesonide prophylaxis group and eight from controls developed clinical FES. There is no statistical significance found between the eventual outcomes of subclinical or clinical FES between the ciclesonide prophylaxis and control group. Although there was a trend seen in the possible preventive efficacy of inhalational steroid in the present study, it did not reach the statistically significant level. The prophylactic role of inhalational steroid in post-traumatic subclinical and clinical FES is statistically insignificant in the present study.

## Introduction

Fat embolism syndrome (FES) is a severe and potentially life-threatening complication of long bone fractures with mortality rates ranging from 10 to 36% [1-2]. The reported incidence of FES in different studies varies from 2% to 22%. As many as 35% to 70% of patients with long bone fractures suffer from subclinical hypoxemia and remain undiagnosed [3]. These patients with clinically inapparent hypoxemia can remain undetected, but may present with clinical FES following surgery for definitive stabilization of fractures [4].

Though embolism of fat globules following long bone fractures is a common event, FES occurs as a result of damage to interstitial and alveolar inflammation triggered by the lipid metabolites in blood circulation. The 'low-dose' corticosteroids given after the skeletal trauma are believed to have a prophylactic effect on the incidence of the fat embolism syndrome and arterial hypoxemia by reducing this inflammatory response [5-6]. However, due to the apprehension of complications with the systemic use of steroids, their use in the prophylaxis of FES is still limited [7]. There is a risk of delayed wound healing and infection in a trauma patient after the steroid therapy.

The aerosol therapy is a modality of drug administration where the main advantage is that for a given therapeutic response, the drug dose is several-fold lower, and the systemic absorption is negligible [8-10]. The aerosol preparations of steroids have the advantages of better pulmonary deposition efficiency, high systemic clearance, and selective binding to the glucocorticoid receptors. The efficacy of inhaled corticosteroids has already been proved in parenchymal lung injury due to chlorine gas [11] and acute respiratory distress syndrome [12].

Ciclesonide is an inhaled corticosteroid administered via a metered-dose inhaler (MDI). It can reach the lung parenchyma, unlike most of the previous steroid inhalers that were unable to go beyond the bronchial tree and had minimal effect on endogenous cortisol. It has already been used successfully in the treatment of allergic rhinitis and asthma.

As FES is primarily a lung parenchyma disorder, it is expected that ciclesonide aerosol given as prophylaxis may be able to stabilize the lung parenchyma. It is also supposed to bear the effects of post-traumatic fat globulinemia and prevent the development of hypoxemia or FES in high-risk patients. To date, there are no published studies on the impact of inhaled steroids in the prophylaxis of FES. Our study was designed to evaluate the role of inhalational corticosteroids in the prevention of fat embolism syndrome in high-risk patients.

## Materials and methods

The study was conducted over a period of two years following the approval from the Institutional Review Board from the Postgraduate Institute of Medical Education and Research (PGIMER), Chandigarh, India. There was no specific approval number assigned at that time. Informed patient consent was obtained prior to treatment.

The selection of patients for the study was made from those attending the surgical emergency of our institute. Patients belonging to the age group of 16-40 years and having a fracture of the femoral shaft alone or associated with other skeletal injuries presenting within eight hours of injury were considered for inclusion. Patients with multisystem trauma (significant head, chest, and abdominal injury), open femoral fractures, pathological fractures, or those with pre-existing cardiac or pulmonary disease or chronic smokers were excluded. After obtaining informed consent, 60 patients of either sex were randomly selected for the study. In all of these patients, after initial resuscitation and necessary splinting, appropriate imaging for all skeletal injuries was done. The investigations at the time of admission included a chest roentgenogram, arterial blood gas analysis (ABG), hemoglobin, hematocrit, platelet count, serum biochemistry, blood glucose levels, serum lactate, and coagulation profile.

The selected patients were put into one of the two groups depending upon their random allocation number. At the earliest possible time after admission to the hospital, the patients allocated to the Group I (n1=30) were given ciclesonide (640 mcg) with a metered dose inhaler. The steroid dose was repeated once again after 24 hours. Patients allocated to Group 2 (n2=30) were not given any steroids and were taken as controls for the study. All the patients, irrespective of the study group, were taken up for surgical stabilization of their fractures as per their conventional management protocol. All patients were monitored for three days irrespective of the intermediate surgical interventions. Complete records were maintained for repeated clinical evaluation, including four hourly vitals (pulse rate, respiratory rate, blood pressure, and temperature) and 12 hourly ABG estimations for 72 hours. During these three days, once daily chest X-ray, hemogram, and coagulation profiles were taken. A note was made of any side effects of the inhalational steroids observed during the hospital admission period, e.g. oropharyngeal candidiasis, any alteration in serum biochemistry, or wound complications.

The outcome was assessed by documentation of any episode of hypoxemia within the 72 hour observation period. The fat embolism index score of all the patients was calculated daily by a blind investigator, based on the Schonfeld, et al. criteria [5]. Petechiae was the most sensitive indicator for FES, followed by hypoxaemia with arterial oxygen partial pressure (pO2 ) lower than 60 mmHg and positive X-ray findings. Patients with subclinical FES were identified as those in whom the score was less than five, but there were episodes of hypoxia diagnosed on serial arterial blood gas estimations. For the diagnosis of subclinical hypoxemia, all patients were classified into six subgroups (three in the control and three in the trial group) according to their pO2 values. We considered hypoxaemia with any pO2 < 70 mmHg and classified all patients in three categories: severe hypoxaemia (pO2 < 60 mmHg), mild hypoxaemia (60 mmHg < pO2 < 70 mmHg), and normal (pO2 > 70 mmHg).

The analysis was done by comparing the incidence of hypoxemia and FES in both groups to determine the effects of prophylactic doses of inhalational steroids. The statistical analysis was done using the ‘Mann-Whitney test’ for quantitative data and Chi-square test for qualitative data. A p-value less than 0.05 were considered to be statistically significant.

## Results

In the first group, there were 22 males and eight females with a mean age of 29 years (range: 18 to 34 years) whereas in control group there were 23 males and seven females with an average age being 28.5 years (range: 20 to 35 years). The variables were compared in each group at the time of admission and further during follow-up. Age, sex, pre-hospital stabilization of fractures with splints, time since the injury on arrival in the hospital, and vital parameters were statistically insignificant between both groups. There was no significant association between the partial pressure of oxygen, hemoglobin, haematocrit, or biochemical parameters at the time of admission and the subsequent development of FES in both the groups.

There were six patients with subclinical FES in each group. Although there were eight cases of clinical FES in the control group and three cases in ciclesonide prophylaxis group, it was statistically insignificant. Thus, the development of FES was not affected by the ciclesonide prophylaxis. Mean values of the arterial blood partial pressure of oxygen are shown in Table 1.

**Table 1:** Mean values of partial pressures of oxygen (in mm Hg) in patients treated with inhaled ciclesonide and controls at 12 hourly intervals since injury.

Mean Values						
Time Since Injury (Hours)	12	24	36	48	60	72
On ciclesonide prophylaxis	90.2	89.7	88.2	85.5	82.2	79.4
Controls	83.4	83.2	81.5	76	75.8	71.6

Clinical observations made in both groups over 72 hours are shown below in Table 2.

**Table 2:** Clinical observations during 72 hours following admission.

Parameter	Ciclesonide Prophylaxis Group	Controls
Petechiae	0	1
Diffuse alveolar infiltrates	3	7
Confusion	1	7
Fever	1	2
Tachycardia	4	9
Tachypnoea	3	7

There was no statistically significant difference in the values of partial pressure of oxygen at 12 hourly intervals during the 72 hours between the two groups.

The fourteen cases of clinical and subclinical FES in the control group and nine cases in the ciclesonide group were followed up for the total duration of episode of hypoxemia, which is defined as arterial partial pressure of oxygen less than 90 mm Hg. The total length of hypoxemia was 70 hours in the ciclesonide group and 79 hours in the control group. This was not found to be statistically significant. One patient in the ciclesonide prophylaxis group and two patients in the control group required ventilatory support. Eventually, all these patients recovered well. There was no mortality in either group.

## Discussion

FES is one of the leading causes of morbidity and mortality following skeletal trauma, particularly in long bone and pelvic fractures [13-16]. Symptoms of FES include a triad of respiratory failure, sensorial disturbances, and petechiae. The majority of mortality is preventable if diagnosed early and treatment is initiated in a timely manner.

Corticosteroids have been extensively studied in the prophylaxis of the FES, but the evidence regarding their efficacy and safety is not strong enough to recommend their routine use [17]. The 'low-dose' corticosteroids given after the skeletal trauma have been seen to have a prophylactic effect on the incidence of the FES and arterial hypoxemia. They mostly act as an anti-inflammatory agent, reducing perivascular hemorrhage and edema resulting from pulmonary parenchymal injury in response to lipid metabolites. Corticosteroid use is primarily limited by the fear of systemic side effects associated with their usage, such as metabolic derangements, delayed wound healing, and increased risk of infection, which are more troublesome for the surgeons. Due to the apprehension of complications with the systemic use of steroids, their use in prophylaxis of FES is still limited.

The aerosol therapy is a modality of drug administration where the main advantage is that for a given therapeutic response, the drug dose is several-fold lower, and the systemic absorption is negligible. However, until a few years ago, the aerosol steroid therapy was effective only up to bronchial level. With the discovery of ciclesonide, which can reach up to the lung parenchyma, the effect can be achieved at the alveolar level. The efficacy of inhaled corticosteroids has already been clinically proved in chlorine gas-induced lung injury and acute respiratory distress syndrome due to sepsis [18-19]. As FES is also primarily a lung parenchyma disorder, it was hypothesized in this study that ciclesonide aerosol given as prophylaxis may be able to stabilize the lung parenchyma and thus able to bear the effects of post-traumatic fat globulinemia. This could prevent the development of hypoxemia or fat embolism syndrome in patients at high risk for this complication. There are still no studies regarding the role of inhaled steroids in the prophylaxis of post-traumatic hypoxia and fat embolism syndrome in the literature.

Ciclesonide is a highly potent and relatively safe inhaled corticosteroid. It also has the advantage of very low oral bioavailability. Inhaled ciclesonide has been shown to be free from systemic side effects when used for management of asthma, even at high doses. Doses of 160 to 640 mcg have been employed in the treatment of bronchial asthma. A ciclesonide dose of 320 mcg is equivalent to 440 mcg fluticasone, the most potent inhaled corticosteroid [20-22]. Out of the 30 patients studied in Group 1, three had a Schonfeld’s score more than or equal to 5, indicating clinical fat embolism syndrome. Six others with subclinical fat embolism syndrome had intermittent episodes of significant hypoxia and their scores were 3-4, whereas, in Group 2, eight patients had clinical fat embolism syndrome and six others had subclinical episodes of hypoxia.

To the best of our knowledge, this is the first study to examine the role of inhaled corticosteroids in the prophylaxis of post-traumatic subclinical and clinical FES. Although there is a biological plausibility that inhaled steroids may be useful, as shown by the study of intravenous steroids in the literature, as well as a trend seen in the possible preventive efficacy of inhalational steroid in the present study, it did not reach a statistically significant level in our study. The possible explanation could be a true inefficacy of the inhalational steroid, the small number of patients in our study, or the effect may be less pronounced than intravenous steroids that the study was not powered to detect. Therefore, larger study groups may be required to define precisely the role of inhalational steroids in the prophylaxis of post-traumatic subclinical and clinical FES. Moreover, as the etiology and pathogenesis of FES is still not certain, there may be other factors also contributing to the above results. Though the number of patients in the present study is small, it is observed that the prophylactic role of inhalational steroids, i.e. ciclesonide, in post-traumatic clinical and subclinical FES is statistically insignificant.

## Conclusions

There is no statistical significance found between the eventual outcomes of subclinical or clinical fat embolism syndrome between the ciclesonide prophylaxis and control groups. Although there was a trend seen in the possible preventive efficacy of inhalational steroid in the present study, it did not reach a statistically significant level.
